# Reliability of the respiratory rate and oxygenation index for successful high-flow nasal cannula support in coronavirus disease pneumonia: a retrospective cohort study

**DOI:** 10.1186/s12890-023-02598-y

**Published:** 2023-08-10

**Authors:** Ryosuke Hirabayashi, Kazuma Nagata, Yuki Sato, Atsushi Nakagawa, Ryo Tachikawa, Hirokazu Kuroda, Ryutaro Seo, Takeshi Morimoto, Keisuke Tomii

**Affiliations:** 1 Department of Respiratory Medicine, Kobe City Medical Center General Hospital, 2-1-1 Minatojima-Minamimachi, Chuo-Ku, Kobe, Hyogo 650-0047 Japan; 2 Department of Infectious Disease, Kobe City Medical Center General Hospital, Kobe, Hyogo Japan; 3 Department of Emergency Medicine, Kobe City Medical Center General Hospital, Kobe, Hyogo Japan; 4 Department of Clinical Epidemiology, Hyogo Medical University, Nishinomiya, Hyogo Japan; 5 Department of Clinical Research Center, Kobe City Medical Center General Hospital, Kobe, Hyogo Japan

**Keywords:** COVID-19, Pneumonia, Respiratory failure, High-flow nasal cannula, ROX index, S/F ratio

## Abstract

**Background:**

High-flow nasal cannula (HFNC) therapy is an important non-invasive respiratory support in acute respiratory failure, including coronavirus disease (COVID-19) pneumonia. Although the respiratory rate and oxygenation (ROX) index is a simple and useful predictor for HFNC failure and mortality, there is limited evidence for its use in patients with COVID-19 pneumonia. We aimed to evaluate the ROX index as a predictor for HFNC failure in patients with COVID-19 pneumonia. We also evaluated the ROX index as a predictor for 28-day mortality.

**Methods:**

In this single-center, retrospective, cohort study, 248 patients older than 18 years of age with COVID-19 pneumonia received HFNC therapy for acute respiratory failure. The ROX index was evaluated within 4 h from the start of HFNC therapy. Past medical history, laboratory data, and the ROX index were evaluated as predictors for HFNC failure and 28-day mortality.

**Results:**

The ROX index < 4.88 showed a significantly high risk ratio for HFNC failure (2.13 [95% confidence interval [CI]: 1.47 – 3.08], *p* < 0.001). The ROX index < 4.88 was significantly associated with 28-day mortality (*p* = 0.049) in patients with COVID-19 pneumonia receiving HFNC therapy. Age, chronic hypertension, high lactate dehydrogenase level, and low ROX index showed significantly high risk ratio for HFNC failure. C-reactive protein level and low ROX index were predictors of 28-day morality.

**Conclusion:**

The ROX index is a useful predictor for HFNC success and 28-day mortality in patients with COVID-19 pneumonia receiving HFNC therapy.

**Trial registration:**

An independent ethics committee approved the study (Research Ethics Review Committee of Kobe City Medical Center General Hospital [number: zn220303; date: February 21, 2022]), which was performed in accordance with the Declaration of Helsinki, Guidelines for Good Clinical Practice.

**Supplementary Information:**

The online version contains supplementary material available at 10.1186/s12890-023-02598-y.

## Introduction

From its first report in December 2019 [1], coronavirus disease (COVID-19) has been an endemic for > 2 years. In Japan, the endemic is still ongoing with approximately 1,720,000 patients and 18,000 deaths during five major epidemics since October 31, 2021. Hypoxemia is one of the most important symptoms and causes of death in moderate-to-severe COVID-19 pneumonia [2], and it requires appropriate respiratory support including invasive or non-invasive ventilatory support.

High-flow nasal cannula (HFNC) therapy is an important non-invasive respiratory support device in acute respiratory failure. It delivers heated and humidified oxygen via the nose at flows as high as 40–60 L/min and at oxygen concentrations up to 100% [3], and it is increasingly being used to support COVID-19 patients with hypoxemic respiratory failure in Japan [4] because of its efficacy in patients and safety for health care providers [5, 6]. Compared with conventional oxygen therapy, HFNC therapy significantly decreases the need for invasive mechanical ventilation and time to clinical recovery in the case of hypoxemic respiratory failure [7]. However, there have been concerns about increased mortality due to the late failure of patients after application of HFNC therapy [8, 9]. Therefore, useful indicators for respiratory failure are important in the management of HFNC therapy.

The respiratory rate and oxygenation (ROX) index, i.e., the ratio of oxygen saturation as measured by pulse oximetry/fraction of inspired oxygen (FiO_2_) to the respiratory rate, is a simple, useful, and well validated tool for predicting the outcome of HFNC therapy (the need for intubation or not and death). Since the ROX index was first reported by Roca et al., the ROX index for HFNC therapy has been discussed from 2 to 24 h after initiation, and its cut-off value is 4.88 ≤ (area under the curve [AUC] 0.74 [0.64–0.84], *p* < 0.002) [10]. However, the S/F ratio (saturation of inspired oxygen [SpO_2_]/fraction of inspired oxygen [FiO_2_] ratio) by itself correlates with the P/F ratio (PaO_2_/FiO_2_ ratio) and is an important indicator of respiratory failure [11]. In COVID-19, the respiratory rate may not indicate severity because the disease is often present with silent hypoxemia, which is associated with an increased respiratory rate [12]; therefore, the S/F ratio is a possible comparable predictive parameter of the ROX index. On the other hands, the presence of silent hypoxemia does not immediately undermine the usefulness of the ROX index, as an observational study showed that even silent hypoxemia is associated with an increased respiratory rate [11]. Several reports have shown the relationship between the ROX index, S/F ratio, and HFNC failure or mortality; yet, the real-world data for HFNC therapy, the ROX index, and S/F ratio in COVID-19 pneumonia are still limited. We hypothesized the reliability of ROX index for HFNC success and failure is still useful, be equal or more than S/F ratio.

Herein, we describe the use of HFNC therapy for patients with moderate to severe COVID-19 pneumonia. The purpose of this study was to investigate the use of HFNC therapy in patients with moderate to severe COVID-19 pneumonia and the factors associated with HFNC failure. We also evaluated 28-day mortality and the factors associated with 28-day mortality.

## Patients and methods

### Study design and population

This single-center, retrospective, cohort study included patients with moderate-to-severe COVID-19 pneumonia from July 20, 2020 to October 31, 2021. Patients at least 18 years or older who required supplemental oxygen were eligible. The following patients were excluded from the study: those who did not require supplemental oxygen and invasive mechanical ventilation, those admitted with other diseases or wounds that did not have a course or clinical findings of COVID-19 pneumonia and were judged clinically not to require oxygen administration due to COVID-19 pneumonia, those who received HFNC therapy for acute respiratory failure with hypercapnia, those who received HFNC therapy as post-extubation support, and those for whom data for the ROX index were not collected within the first 4 h using HFNC therapy. We also excluded patients with do-not-intubate orders on hospital admission.

### Data collection

Patients’ characteristics, medical history, laboratory examination data, imaging findings, treatment, outcome, and complications were collected from the medical records. All patients were diagnosed with COVID-19 based on a severe acute respiratory syndrome coronavirus 2-specific polymerase chain reaction test result obtained from a nasal or oropharyngeal swab. A chest X-ray or chest computed tomography scan was evaluated to detect bilateral consolidation or ground-glass opacity, which was indicative of pneumonia. All treatments followed the Japanese guideline for COVID-19 or Infectious Diseases Society of American guideline for COVID-19 at the time and included a systemic glucocorticoid (e.g., dexamethasone, methylprednisolone, and hydrocortisone), remdesivir, tocilizumab, baricitinib, and unfractionated heparin.

### Respiratory support and outcomes

We used Optiflow™ (Fisher and Paykel Healthcare) as the HFNC device for COVID-19 pneumonia. HFNC therapy was considered for the patients who needed > 5 L/min of supplemental oxygen and a respiratory ratio of > 25 /min, and those who expected to avoid intubation management with the introduction of HFNC. HFNC therapy was started and fixed at a 40-L/min flow and had an appropriate FiO_2_. Patients discontinued HFNC therapy at the discretion of the attending physician or intensive care unit doctor based on an FiO_2_ of 0.35–0.40 and clinical improvement. All patients except those who refused intubation were considered for intubation and mechanically ventilation at a P/F ratio < 70–100 with clinical worsening. Intubation and mechanical ventilation were initiated at the discretion of the attending physicians and intensive care specialists when patients or their family members refused the do-not-intubate order based on the physicians' opinion of the patient's condition. Specialists followed the mechanical ventilation protocol at our institution with reference to the criteria used in previous studies. For example, the following criteria were used for initiating invasive ventilation: hemodynamic instability, deterioration of neurologic status, or signs of persisting or worsening respiratory failure. This was defined by at least two of the following criteria: respiratory rate of > 40 breaths per minute, lack of improvement in signs of high respiratory muscle workload, and copious tracheal secretions. Other criteria included acidosis with a pH of < 7.35 and a percutaneous arterial oxygen saturation of < 90% for > 5 min without technical dysfunction or poor response to oxygenation techniques. Non-invasive positive-pressure ventilation was not used for acute respiratory failure to ensure infection control for the medical staff.

The ROX index [10] and S/F ratio were measured based on the patient’s vital signs (saturation of inspired oxygen [SpO_2_] and respiratory rate) and FiO_2_. Because these vital signs were not routinely measured immediately after HFNC initiation, we adopted the ROX index as early as possible after HFNC initiation; the acceptable range was within 4 h.

We defined the primary outcome for this study as HFNC failure, which means intubation or death with HFNC therapy. We defined the secondary outcome as 28-day hospital mortality, which is the death within 28 days in our hospital, with HFNC use and with or without intubation. The cause of death was not considered in this study. We also compared the reliability of the ROX index and S/F ratio as the predictor of HFNC success or failure and 28-days mortality in our cohort.

### Cut-off values and statistical analysis

Based on the risk factors for severity and death on hospital admission for Japanese patients with COVID-19 pneumonia [12], we evaluated the followed factors: older age (> 65 years), higher body mass index (BMI, > 30 kg/m^2^), cardiovascular disease, cerebrovascular disease, chronic obstructive pulmonary disease (COPD), diabetes mellitus, solid tumor, and hypertension. We also considered past or current smoking status [13], C-reactive protein (CRP) level > 10.0 mg/dL (cut-off value, 9.7–11 mg/dL [14, 15]), and lactate dehydrogenase (LDH) level > 450 IU/L (cut-off value, 245–450 U/L [15, 16]) as the risk factors for respiratory failure or death. We defined the cut-off value for the ROX index as < 4.88, according to the original study [10].

Continuous variables are reported as median and interquartile range (IQR), and categorical variables are expressed as number and percentage. Comparisons between the HFNC failure and success groups were performed using the Wilcoxon rank-sum test for continuous variables and the Fisher exact test for categorical variables. To analyze the primary and secondary outcome, we used a Cox regression model adjusted for older age (> 65 years), higher BMI (> 30 kg/m^2^), cardiovascular disease, cerebrovascular disease, COPD, diabetes mellitus, solid tumor, hypertension, past or current smoking status, CRP level > 10.0 mg/dL, LDH level > 450 IU/L, and ROX index < 4.88. HFNC failure were compared between the ROX ≥ 4.88 and < 4.88. For the HFNC failure group, 28-day mortality was compared between these groups. We used the log-rank test and the Kaplan–Meier Method to perform survival analysis. Based on our cohort, we created an ROC curve and examined cut-off values by Youden index. Missing values were found at a level of < 10% of the total, and we did not perform imputation. We used JMP, version 16.0 (JMP Statistical Discovery LLC) to perform the statistical analyses. A 2-tailed *p*-value of < 0.05 was considered statistically significant.

## Results

**Fig. 1:**
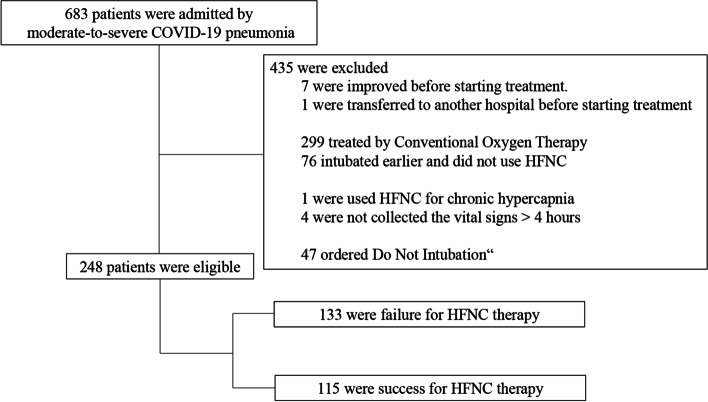
Trial profile

Among the 683 patients, 435 were excluded for the following reasons; transferred to another hospital before starting treatment, improved and did not need treatment, received HFNC therapy for chronic hypercapnia, missing vital signs and scores, and had do-not-intubate orders. Therefore, 248 patients were included in this study (Fig. 1). All patients received HFNC therapy and mechanical ventilation without facility restrictions.

Table 1 shows the patient characteristics. In the HFNC success group, 6 patients had missing data for the BMI and 1 patient had missing data for the serum albumin level. In the failure group, 2 patients had missing data for the BMI, 6 for the lymphocyte count, and 2 for the LDH level. The HFNC failure group included more patients with older age, COPD, and diabetes mellitus than the HFNC success group. Overall, 133 patients failed HFNC therapy. HFNC failure patients had a higher respiratory rate, lower lymphocyte count, and higher LDH and creatinine levels. There were no significant differences between the two groups; the smoking history, frequency of cardiovascular disease or hypertension, albumin level, and CRP level.

**Table 1:** Patient characteristics

Variable	HFNC success (*n* = 115)	HFNC failure (*n* = 133)	*P* value
Age, y	57 [52–70]	64 [55–71.5]	0.016
Sex, male (%)	87 (75.7)	103 (77.4)	0.74
BMI, kg/m^2^	25.8 ± 4.6	26.1 ± 5.1	0.67
Smoking history, past or current (%)	76 (66.1)	86 (65.3)	0.81
Past medical history, number (%)			
Asthma	10 (8.7)	9 (6.8)	0.57
COPD	4 (3.5)	28 (13.5)	0.004
Interstitial Pneumonia	0 (0)	3 (2.3)	0.52
Malignancy	9 (7.8)	10 (7.5)	0.93
HIV infection, AIDS	0 (0)	1 (0.8)	0.26
Diabetes mellitus	23 (20.0)	43 (32.3)	0.03
Chronic kidney disease	14 (12.2)	9 (6.8)	0.14
Hemodialysis	1 (0.9)	1 (0.8)	0.91
Cardiovascular disease	13 (11.3)	22 (16.5)	0.23
Hypertension	60 (52.2)	65 (48.9)	0.60
Chronic liver disease	7 (6.1)	12 (9.0)	0.38
Cerebrovascular disease	8 (7.0)	12 (9.0)	0.55
Vital signs on admission			
Mean arterial pressure	101.2 ± 14.0	99.8 ± 13.0	0.44
Heart rate	89 ± 17	90 ± 18	0.63
Respiratory rate	24 ± 6	27 ± 7	0.03
Consciousness disorder (GCS score < 15), (%)	8 (7.0)	17 (12.9)	0.11
Laboratory test results			
White blood cell count, × 10^3^/µL	7.4 [5.2–10.4]	6.5 [4.65–9.4]	0.08
Lymphocyte count, /µL	608 [426–840]	487 [350–730]	0.01
Hct level, %	41.1 ± 4.2	40.3 ± 5.3	0.63
Serum albumin level, g/dL	3.0 ± 0.4	2.9 ± 0.4	0.39
Lactate dehydrogenase level, IU/L	423 [345–548]	521 [441–662]	< 0.0001
Na level, mEq/L	135.8 ± 4.3	135.6 ± 4.4	0.90
K level, mEq/L	4.08 ± 0.56	4.08 ± 0.56	0.73
Creatinine level, mg/dL	0.75 [0.63–0.98]	0.83 [0.67–1.05]	0.07
C-reactive protein level, mg/dL	8.24 [4.68–12.78]	9.19 [5.045–12.495]	0.73

*BMI* Body mass index, *COPD* Chronic obstructive pulmonary disease, *HIV* Human immunodeficiency virus, *AIDS* Acquired immunodeficiency syndrome, *GCS* Glasgow Coma Scale, *Hct* Hematocrit, *HFNC* High-flow nasal cannula

Table 2 shows the intervention including respiratory support by HFNC and complications. All patients received systemic corticosteroids (6 mg of dexamethasone or an equivalent dose of methylprednisolone or hydrocortisone, for at least 10 days). The HFNC flow was fixed at 40 L/min. There was no significant difference in adjunctive treatment between the HFNC success and failure groups. Major complications were gastroenteric bleeding, hospital-acquired pneumonia, and bacteremia. All cases of gastroenteric bleeding were not severe or fatal. Compared to the HFNC success group, the HFNC failure group had a shorter time from hospital admission to the start of HFNC therapy, higher respiratory rate, lower S/F ratio, and lower ROX index. The numbers of patients with an ROX index ≥ 4.88 were 97 (84.4%) in the HFNC success group and 84 (63.2%) in the failure group. Because of delayed implementation, 17.7% of patients were treated in the awake prone position (HFNC success group: 20%; HFNC failure group: 15.8%), but there was no statistically significant difference between the groups.

**Table 2:** Intervention, respiratory support, parameters, and complications

Variable	HFNC success (*n* = 115)	HFNC failure (*n* = 133)	*P* value
Treatment, number (%)			
Systemic corticosteroids	115 (100)	133 (100)	
Remdesivir	76 (66.1)	72 (54.1)	0.06
Tocilizumab	101 (84.2)	110 (77.2)	0.26
Complication			
Gastroenteric bleeding (%)	1 (0.9)	8 (6.0)	0.02
Hospital-acquired pneumonia (%)	9 (7.8)	42 (31.6)	< 0.0001
Bacteremia (%)	3 (2.6)	19 (14.3)	0.0006
Length of hospital stay, day [IQR]	13 [11–19]	25 [17.5–42.5]	< 0.0001
28-day mortality, number (%)		16 (12.0)	
HFNC and respiratory support			
HFNC flow, 40 L/min, number (%)	115 (100)	133 (100)	
Admission to start HFNC use, days	1 [0–2]	0 [0–0]	< 0.0001
RR after starting HFNC, /min	22 ± 6	25 ± 6	0.002
S/F ratio after starting HFNC	157.2 ± 32.6	137.6 ± 35.2	< 0.0001
ROX index after starting HFNC	7.68 ± 3.01	6.04 ± 2.49	< 0.0001
ROX index < 4.88, number (%)	18 (15.7)	49 (36.8)	0.0001
ICU stay, number (%)	75 (65.2)	133 (100)	
Awake prone position, number (%)	23 (20.0)	21 (15.8)	0.38
Intubation and MV, number (%)		133 (100)	

*IQR* Interquartile range, *HFNC* High-flow nasal cannula, *RR* Respiratory ratio, *S/F ratio* Saturation of inspired oxygen/fraction of inspired oxygen ratio, *ROX index* Respiratory rate and oxygenation index, *ICU* Intensive care unit, *MV* Mechanical ventilation

**Fig. 2:**
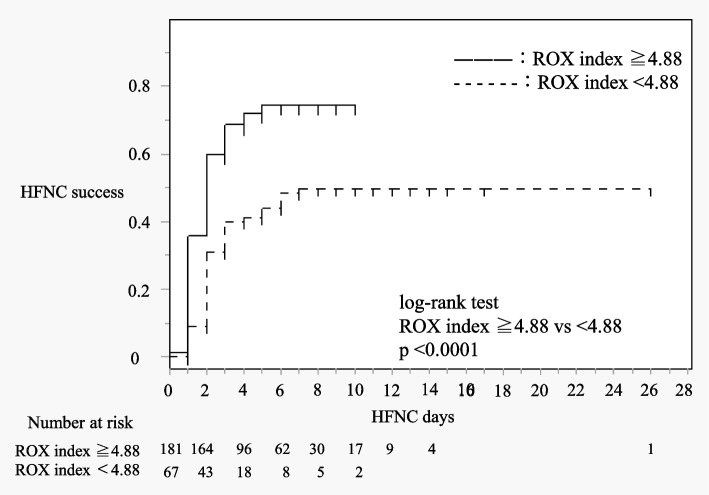
log-rank test and Kaplan-Mayer curve for HFNC failure

Table 3 shows the result of multivariate analysis of the predictors for HFNC success versus HFNC failure. Figure 2 shows log-rank test and Kaplan-Mayer curve for HFNC failure between ROX index < 4.88. The predictors of HFNC failure were as follows: age ≥ 65 years, hypertension, LDH level > 450 U/L, and ROX index < 4.88. The time to event is shown in Fig. 2.

**Table 3:** Predictors of HFNC failure

Variable	Risk ratio (95% CI)	*P* value
Age, ≥ 65 y	1.49 (1.02–2.16)	0.04
BMI, > 30 kg/m^2^	1.36 (0.84–2.20)	0.21
Smoking status	0.97 (0.66–1.42)	0.87
COPD	1.60 (0.93–2.77)	0.09
Malignancy	0.83 (0.43–1.62)	0.83
Cardiovascular disease	1.46 (0.90–2.37)	0.12
Cerebrovascular disease	1.27 (0.69–2.36)	0.44
Diabetes mellitus	1.31 (0.90–1.91)	0.15
Hypertension	0.69 (0.48–0.9989)	0.049
LDH level, > 450 U/L	1.72 (1.16–2.53)	0.006
CRP level, > 10 g/dL	1.05 (0.73–1.49)	0.81
ROX index, < 4.88	2.13 (1.47–3.06)	< 0.001

*HFNC* High-flow nasal cannula, *BMI* Body mass index, *COPD* Chronic obstructive pulmonary disease, *LDH* Lactate dehydrogenase, *CRP* C-reactive protein, *ROX index* Respiratory rate and oxygenation index, *CI* Confidence interval

**Fig. 3:**
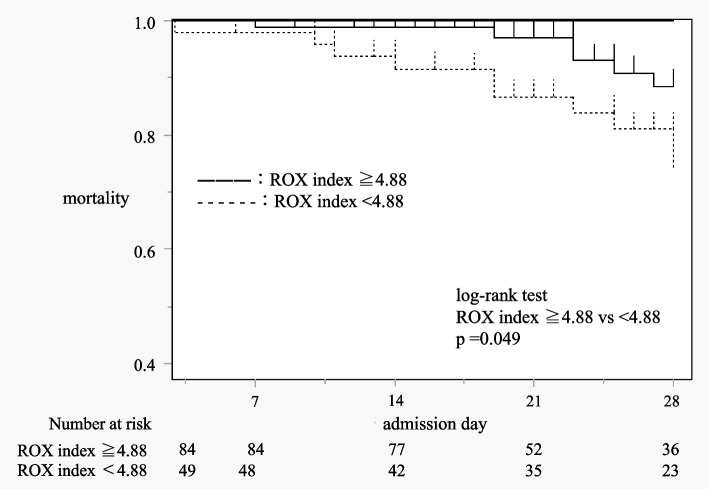
log-rank test and Kaplan-Mayer curve for 28-day mortality in HFNC failure patients

Table 4 shows results of the analysis of 28-day mortality for HFNC failure, adjusted by intubated patients. Figure 3 shows the log-rank test and Kaplan-Mayer curve for 28-day mortality in HFNC failure patients. In univariate analysis, hypertension and ROX index are significantly different (Table 4). In multivariate analysis, ROX index < 4.88 and low CRP level were independent predictors of 28-day mortality (Table 5). The time to event is shown in Fig. 3.

**Table 4:** Predictors of 28-day mortality adjusted for patients with HFNC failure, univariate analysis

Variable	Survive, *N* = 117	Death, *N* = 16	*P* value
Age, years old	64 [55.5–72]	68 [53.25–71]	0.84
BMI, kg/m^2^	25.9 ± 5.0	27.7 ± 5.6	0.11
Smoking status, past or current (%)	75 (64.1)	11 (68.8)	0.79
COPD	16 (13.7)	2 (12.5)	1.00
Malignancy	8 (6.8)	2 (12.5)	0.34
Cardiovascular disease	19 (16.2)	3 (18.8)	0.73
Cerebrovascular disease	10 (8.6)	2 (12.5)	0.64
Diabetes mellitus	35 (29.9)	8 (50.0)	0.15
Hypertension	53 (45.3)	12 (75.0)	0.03
LDH level, U/L	519 [436–655]	613.5 [464.75–793.5]	0.20
CRP level, g/dL	9.35 [5.25–12.50]	5.75 [3.11–12.57]	0.18
ROX index	6.20 ± 2.51	4.89 ± 2.04	0.04

*HFNC* High-flow nasal cannula, *BMI *Body mass index, *COPD *Chronic obstructive pulmonary disease, *LDH *Lactate dehydrogenase, *CRP *C-reactive protein, *ROX index *Respiratory rate and oxygenation index, *CI *Confidence interval

**Table 5:** Predictors of 28-day mortality adjusted for patients with HFNC failure, multivariate analysis

Variable	Risk ratio (95% CI)	*P* value
Age, ≥ 65 y	1.74 (0.50–6.07)	0.38
BMI, > 30 kg/m^2^	2.50 (0.63–9.94)	0.19
Smoking status	1.58 (0.46–5.41)	0.49
COPD	0.95 (0.19–4.88)	0.95
Malignancy	1.08 (0.21–5.61)	0.92
Cardiovascular disease	1.29 (0.30–5.57)	0.73
Cerebrovascular disease	1.72 (0.34–8.79)	0.51
Diabetes mellitus	2.75 (0.93–8.14)	0.07
Hypertension	1.91 (0.54–6.72)	0.31
LDH level, > 450 U/L	1.74 (0.43–6.98)	0.43
CRP level, > 10 g/dL	0.27 (0.08–0.99)	0.048
ROX index, < 4.88	3.59 (1.17–11.08)	0.026

*HFNC* High-flow nasal cannula, *BMI* Body mass index, *COPD* Chronic obstructive pulmonary disease, *LDH* Lactate dehydrogenase, *CRP* C-reactive protein, *ROX index* Respiratory rate and oxygenation index, *CI* Confidence interval

Supplementary table shows the sensitivity, specificity, positive predictive value (PPV), negative predictive value (NPV), and positive/negative likelihood ratio (LR ±) for HFNC success or failure and 28-day mortality among patients with an ROX index < 4.88 in this study. Supplementary figure shows the receiver operating characteristic curves for the S/F ratio and ROX index in this cohort. The cut-off value for HFNC failure in this cohort was an ROX index of 6.57 and S/F ratio of 134.29, with AUCs of 0.67 (*p* ≤ 0.0001) and 0.67 (*p* ≤ 0.0001), respectively. The cut-off value for mortality was an ROX index of 5.28 and S/F ratio of 95.0, with AUCs of 0.72 (*p* = 0.0009) and 0.68 (*p* = 0.005), respectively. (These table and figure are in supplemental material).

## Discussion

### Main findings

This was a single-center, large retrospective study of HFNC therapy for COVID-19 in Japan. Patients with HFNC failure were significantly older, had more COPD or diabetes mellitus, had a worse respiratory condition, and had a higher LDH level than those with HFNC success. A ROX index of < 4.88 within 4 h of starting HFNC therapy was significant indicator of HFNC failure and 28-day mortality. For instance, the predictive value of this cut-off index was poor, especially in sensitivity. HFNC failure was correlated not only with the ROX index but also with various background diseases and blood test results at hospital admission. Twenty-eight-day mortality was significantly associated with an ROX index of < 4.88 and the CRP level.

As shown in Table 3, it is difficult to predict HFNC failure based on the ROX index; the cut-off value of 4.88 for the ROX index is a predictive value for avoidance of intubation, and the cut-off value leading to intubation is lower. Therefore, the present results are consistent with those of a previous report [10]. The association of low CRP levels with mortality is paradoxical. This study included patients who were transported while being treated at another facility, and prior treatment may have influenced the results.

### ROX index and HFNC failure or mortality

In our study, the ROX index predicted HFNC failure well. The ROX index has been shown to be associated with HFNC failure in several reports on COVID-19 pneumonia [17–21]. The variation between our findings and those from these previous reports is attributed to the optimal results for each cohort, but we believe that the first assessment of the ROX index within 4 h can be an indicator of HFNC failure. A large validation study is needed to evaluate this point in more detail. In the latest meta-analysis of HFNC use and the ROX index, the sensitivity, specificity, and AUC of the ROX index were 0.70, 0.79, and 0.81, respectively [22]. In the current study, the ROX index thresholds was variable compared to the original ROX index study [10] or this meta-analysis, and its sensitivity and specificity were not high. These variabilities can be attributed to the patients’ severity, background disease, or characteristics. The current study included very severe patients compared to the cohort of the original study, and the patient characteristics were different between the studies. The selection and use of antiviral drugs, immunosuppressants, and steroids, as well as the response to these drugs, may also be attributable to this variability.

Based on our results, 28-day mortality did not show many associated factors compared to HFNC failure; only the CRP level and ROX index were significant factors for 28-day mortality. Few reports have studied the ROX index and mortality [23, 24]. Interestingly, Kljakovic Gaspic et al. examined in-hospital mortality scoring for patients with HFNC therapy [24], with the following requirements: age ≥ 65 years, ROX index ≤ 4.11, LDH-to-white blood cell count ratio, and Charlson Comorbidity Index as the index for past medical history. In this scoring, the ROX index is a major factor compared to the other requirements for scoring. The ROX index itself plays a greater role in in-hospital mortality than underlying disease, age, or laboratory data. This may be reflected in the results of the present study. However, the mortality rate is affected by the treatment choice and quality of intensive care.

A previous study showed an association with the severity of acute respiratory distress syndrome (ARDS) and mortality in COVID-19 pneumonia [25]. A lower P/F ratio, which means poor oxygenation, is a poor prognostic factor in patients with COVID-19 [26, 27]. The respiratory rate is also associated with mortality in ARDS [28–30]. In the early phases of ARDS, before a patient has fatigued or been sedated, the high transpulmonary pressures associated with spontaneous vigorous inspiratory effort may contribute to damage (so-called patient self-induced lung injury [P-SILI]) [31]. The ROX index is based on SpO_2_/FiO_2_ and the respiratory rate, each of which reflects very poor oxygenation and higher respiratory drive. In particular, a worsening respiratory rate may not only be affected by P-SILI but also by respiratory muscle fatigue [32], which may affect respiratory failure and prognosis. Thus, these composite outcomes may more clearly define HFNC failure or mortality.

Several reports have examined various factors in the study of hospital mortality due to COVID-19 pneumonia [21–23], and other reports have shown the usefulness of existing composite outcomes such as the Acute Physiology and Chronic Health Evaluation (APACHE) II and Sequential Organ Failure Assessment (SOFA) scores [16, 19]. The data collection criteria such as the hospital admission date, inclusion criteria, or intubation also differ. Considering these findings, a large number of factors can be associated with mortality in terms of items and time. The present study only examined past medical history, some laboratory data, and the ROX index, so the selection and use of antiviral drugs, immunosuppressants, and steroids, as well as the response to these drugs, may be attributable to this variability. Further examination with larger sample sizes and adequate data on hospital admission and intubation may yield more accurate results.

### ROX index and S/F ratio

In the present study, both the S/F ratio and ROX index were shown to be useful as predictors of HFNC failure and in-hospital mortality. Kim et al. already showed that the S/F ratio at the time of HFNC initiation is an acceptable predictor of HFNC failure [33], and the results of this study were similar, indicating that the S/F ratio is also an acceptable predictor for in-hospital mortality.

Our study did not compare the SF ratio and ROX index in terms of their usefulness. The S/F ratio is a simple index, it can be more easily measured, and it may reduce contact between patients and health care workers because it does not require a direct contact to patients. Therefore, it is necessary to evaluate each of these parameters and to use them appropriately depending on the situation. The external validity of the ROX index has been studied more than that of the S/F ratio, and we proposed a cut-off value of 4.88 for the ROX index; therefore, it can be used for risk assessment. Although the S/F ratio has not been well validated in COVID-19, it can be considered as an useful index, as its easy measurement characteristics are one of its advantages. In present study, cut-off values for both parameters were retrieved and their usefulness was presented because both of them have usefulness that should be utilized.

### Strengths

Although the ROX index can be related to the risk of 28-day mortality, there have been few reports about the risk. Herein, we showed that the ROX index at the start of HFNC therapy is a predictive factor for 28-day mortality. This result may be due to the disease severity and proportion of HFNC failures in this cohort (more than half of patients were considered a failure) compared to cohorts in previous reports.

### Limitations

There are several limitations to this study. This study was a single-center, retrospective study with a small number of missing data. Not all tests were performed on hospital admission, and factors such as the partial pressure of oxygen level, procalcitonin level, D-dimer level, SOFA score, and APACHE II score were not considered. This study did not fully examine the background of treatment changes and epidemic variants during the study period. However, the omicron variant, which is the mainstay of the current infection epidemic but is not as severe as the delta variant, was not reported in Japan during the study period. The ROX index could not be measured immediately after HFNC initiation in some cases, which may bias the data.

## Conclusion

This study showed that the ROX index is an important predictive factor for both HFNC success and 28-day mortality, making it useful information for clinical management.

### Supplementary Information

**Additional file 1: Supplementary table.** Accuracy, PPV, NPV, and LR values of the ROX index.

**Additional file 2.** The receiver operating characteristic curves for the S/F ratio and ROX index in this cohort.

## Data Availability

The datasets used and/or analyzed during the current study are available from the corresponding author on reasonable request.

## Abbreviations

APACHE II: Acute Physiology and Chronic Health Evaluation II
ARDS: Acute respiratory distress syndrome
BMI: Body mass index
COVID-19: Coronavirus disease
COPD: Chronic obstructive pulmonary disease
CRP: C-reactive protein
FiO2: Fraction of inspired oxygen
HFNC: High-flow nasal cannula
IQR: Interquartile range
LDH: Lactate dehydrogenase
PaO_2_: Arterial partial pressure of oxygen
P-SILI: Patient self-induced lung injury
ROX index: Respiratory rate and oxygenation index
SOFA: Sequential Organ Failure Assessment
SpO_2_: Saturation of inspired oxygen
vs.: Versus
OR: Odds ratio
