# Supplementary material for: Reliability of the respiratory rate and oxygenation index for successful high-flow nasal cannula support in coronavirus disease pneumonia: a retrospective cohort study
Source: BMC Pulm Med. 2023 Aug 10;23:294. doi: 10.1186/s12890-023-02598-y (PMC10413522; doi:10.1186/s12890-023-02598-y)
Supplement: Supplementary file 1 — Additional file 1: Supplementary table. Accuracy, PPV, NPV, and LR values of the ROX index. [file 12890_2023_2598_MOESM1_ESM.doc]

| Supplementary table. Accuracy, PPV, NPV, and LR values of the ROX index | | | | | | |
| --- | --- | --- | --- | --- | --- | --- |
|  | Sensitivity, % | Specificity, % | PPV, % | NPV, % | LR+ | LR- |
|  |  |  |  |  |  |  |
| HFNC failure, ROX index <4.88 | 36.8 [31.8–41.1] | 84.3 [78.5–89.2] | 73.1 [63.2–81.5] | 53.6 [49.9–56.7] | 2.35 [1.48–3.81] | 0.75 [0.66–0.87] |
| 28-day mortality, ROX index <4.88 | 62.5 [39.5–81.1] | 75.4 [73.8–76.7] | 14.9 [9.4–19.4] | 96.7 [94.7–98.3] | 2.54 [1.51–3.48] | 0.50 [0.25–0.82] |
|  |  |  |  |  |  |  |
| HFNC: high-flow nasal cannula, ROX index: respiratory rate and oxygenation index, PPV: positive predictive value, NPV: negative predictive value, LR+: positive likelihood ratio, LR-: negative likelihood ratio | | | | | | |
