# Supplementary material for: Reliability of the respiratory rate and oxygenation index for successful high-flow nasal cannula support in coronavirus disease pneumonia: a retrospective cohort study
Source: BMC Pulm Med. 2023 Aug 10;23:294. doi: 10.1186/s12890-023-02598-y (PMC10413522; doi:10.1186/s12890-023-02598-y)
Supplement: Supplementary file 2 — Additional file 2. The receiver operating characteristic curves for the S/F ratio and ROX index in this cohort. [file 12890_2023_2598_MOESM2_ESM.pdf]

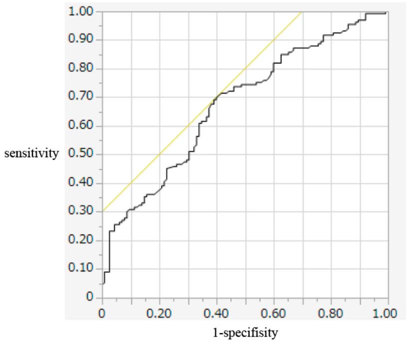

ROX index
for HFNC failure

cut off: 6.57
AUC: 0.67
Se: 69.2% Sp: 60.9%
p=<0.0001

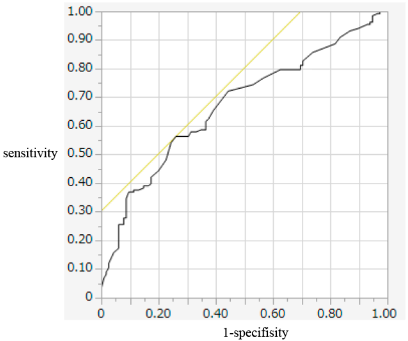

S/F ratio
for HFNC failure

cut off: 134.29
AUC: 0.67
Se: 56.4% Sp: 73.9%
p=<0.0001

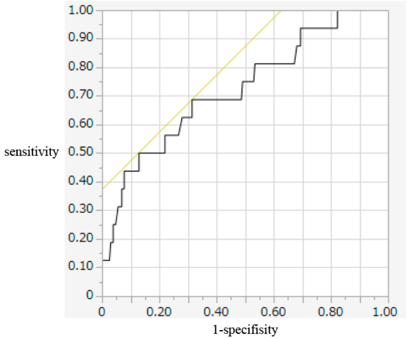

ROX index
for 28-day mortality

cut off: 5.28
AUC: 0.72
Se: 68.8% Sp: 68.5%
p=0.0009

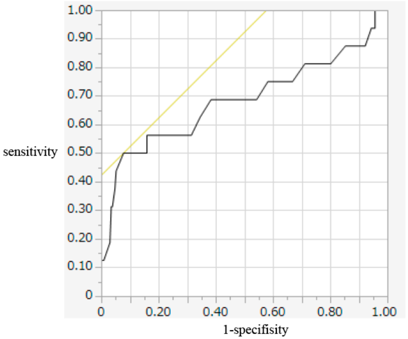

S/F ratio
for mortality

cut off: 95.0
AUC: 0.68
Se: 50% Sp: 92.2%
p=0.005
